# Clinical Feasibility and Skeletal Effects of Digitally Guided Supragingival Miniplates for Herbst Therapy in Late Adolescents: A Pilot Study

**DOI:** 10.3390/jcm15083059

**Published:** 2026-04-16

**Authors:** Ignasi Arcos, Andre Walter, Théophile Marc, Luis Carlos Ojeda, Andreu Puigdollers

**Affiliations:** 1 Departament d’Ortodoncia, Facultat d’Odontologia, Universitat Internacional de Catalunya, C/Josep Trueta s/n, 08195 Barcelona, Spain; 2 Clinica Ojeda Perestelo, C/Peregrina 14, 35002 Las Palmas de Gran Canaria, Spain

**Keywords:** Class II malocclusion, Herbst appliance, skeletal anchorage, supragingival miniplates, miniscrews, digital workflow, guided surgery, mandibular advancement

## Abstract

**Background**: Conventional Herbst appliances are effective for the correction of skeletal Class II malocclusion, but they are frequently associated with dentoalveolar side effects, particularly lower incisor proclination. Skeletal anchorage systems may improve orthopedic outcomes; however, submucosal miniplates require invasive surgical procedures that may reduce patient acceptance. This pilot clinical study evaluated the feasibility, safety, and skeletal effects of a minimally invasive digitally guided protocol using supragingival miniplates for bone-supported Herbst therapy in late adolescents. **Methods**: Eleven late-adolescent patients (14–17 years; cervical vertebral maturation stages CS4–CS5) with skeletal Class II malocclusion due to mandibular retrusion were prospectively treated using a bone-supported Herbst appliance anchored to digitally planned supragingival stainless-steel miniplates fixed with bicortical miniscrews. Miniscrew placement was planned by merging CBCT and intraoral scan data and performed using 3D-printed surgical guides. Cephalometric variables, including SNA, SNB, Wits appraisal, mandibular plane angle, and incisor inclinations, were assessed before treatment and after a 10-month Herbst phase. Mandibular advancement was additionally explored using a complementary linear measurement (SeMndb-line). **Results**: All patients completed treatment without anchorage loss, appliance failure, or surgical complications. Significant skeletal improvements were observed, including an increase in SNB (+3.36°, *p* < 0.001) and a reduction in Wits appraisal (−2.65 mm, *p* < 0.001). The SeMndb-line increased by +3.49 mm (*p* < 0.001), supporting effective mandibular advancement. Lower incisor inclination remained stable (Δ = −0.18°, *p* = 0.909), indicating effective dentoalveolar control. No clinically relevant changes in vertical skeletal pattern were observed. **Conclusions**: Digitally guided supragingival miniplates for bone-supported Herbst therapy appear to be a feasible and minimally invasive approach for the treatment of skeletal Class II malocclusion in late adolescents. This protocol achieved clinically meaningful mandibular advancement while minimizing dentoalveolar side effects. Given the pilot design, small sample size, and lack of a control group, further controlled studies with larger samples and long-term follow-up are required.

## 1. Introduction

Skeletal Class II malocclusion due to mandibular retrusion is a common orthodontic condition that may compromise facial esthetics, occlusal function, and long-term stability. Functional appliances have long been used to correct this discrepancy by promoting mandibular advancement during growth. Among fixed functional devices, the Herbst appliance remains one of the most widely used and clinically effective options, as it does not depend on patient compliance and provides consistent sagittal correction [1,2].

However, conventional dentally anchored Herbst mechanics are frequently associated with significant dentoalveolar side effects, particularly lower incisor proclination and mandibular anchorage loss. These effects tend to be more pronounced in patients approaching the end of their pubertal growth period, where the orthopedic component of correction may be partially masked by dental compensation [3]. In addition, appliance-related complications and maintenance requirements remain relevant clinical limitations [4].

The introduction of skeletal anchorage has expanded the biomechanical possibilities of Class II correction. Previous studies have shown that the use of miniscrews in combination with Herbst appliances can reduce dentoalveolar side effects and enhance skeletal outcomes by improving force transmission to the basal bone [5,6]. Nevertheless, most bone-anchored Herbst protocols rely on submucosal miniplates that require surgical flap elevation, which increases surgical morbidity, postoperative discomfort, and the risk of soft-tissue complications, potentially limiting patient acceptance, particularly in adolescent populations [7,8,9].

Recent advances in digital orthodontics have introduced new possibilities to improve the precision and predictability of skeletal anchorage placement. The integration of cone-beam computed tomography and intraoral scanning allows detailed three-dimensional assessment of bone anatomy, while computer-assisted planning and 3D-printed surgical guides facilitate accurate miniscrew positioning and reduce operator-dependent variability [10,11].

Within this context, there is a clinical need for minimally invasive bone-supported functional protocols that combine effective skeletal anchorage with reduced surgical morbidity and improved procedural precision. A supragingival miniplate system, inserted using a fully digital guided workflow, may represent a promising alternative to conventional submucosal approaches.

Therefore, the aim of this pilot clinical study was to evaluate the feasibility, safety, and cephalometric effects of a digitally guided supragingival bone-supported Herbst protocol in late-adolescent patients with skeletal Class II malocclusion, with particular emphasis on mandibular advancement and control of dentoalveolar side effects.

The primary objective of the study was to evaluate skeletal changes associated with mandibular advancement using a supragingival bone-supported Herbst appliance. Secondary objectives included assessment of dentoalveolar effects, anchorage stability, and the clinical feasibility of the protocol.

## 2. Materials and Methods

### 2.1. Study Design and Ethical Approval

This prospective pilot clinical study was conducted in accordance with the Declaration of Helsinki and was approved by the Ethics and Drugs Committee of Universitat Internacional de Catalunya under protocol number ORT-ECL-2025-04. Written informed consent was obtained from all patients and their legal guardians prior to inclusion.

Eleven consecutive late-adolescent patients (6 females and 5 males; mean age 15.5 ± 1.2 years, range 14–17 years) presenting skeletal Class II malocclusion due to mandibular retrusion were included. Skeletal maturation stage was assessed using the cervical vertebral maturation (CVM) method, and all patients were classified as CS4 or CS5 [12]. All patients were treated at the Department of Orthodontics, Universitat Internacional de Catalunya, Barcelona, Spain.

Inclusion criteria were skeletal Class II malocclusion of mandibular origin with an overjet between 7 and 11 mm; age between 14 and 17 years at treatment onset; adequate bone quality for miniscrew placement (D1 or D2 according to Misch classification) [13,14]; minimum palatal bone height ≥ 6 mm and mandibular transalveolar depth ≥ 7 mm; interradicular space ≥ 3.8 mm at planned mandibular insertion sites; and absence of systemic or debilitating disease.

Additionally, all patients were required to present a skeletal Class II pattern primarily due to mandibular retrusion, confirmed by cephalometric parameters, including an ANB angle > 4°, a reduced SNB angle, and a Wits appraisal > 2 mm. Patients with Class II malocclusion predominantly due to maxillary prognathism or dentoalveolar compensation were not included.

Exclusion criteria included age < 14 years or >17 years at treatment onset, overjet > 13 mm, insufficient bone quantity or density (D3–D4), ongoing medication affecting bone metabolism, or contraindications for orthodontic miniscrew placement.

As a pilot study, no formal sample size calculation was performed; the study was designed to explore feasibility, safety, and preliminary effect sizes to inform future controlled trials.

### 2.2. Digital Records and Planning

All patients underwent cone-beam computed tomography (CBCT; CS 8200, Carestream Dental, Atlanta, GA, USA) and intraoral scanning before treatment. DICOM files obtained from CBCT and STL files from intraoral scans were merged using dedicated digital planning software to generate a three-dimensional anatomical model for each patient.

**Figure 1:**
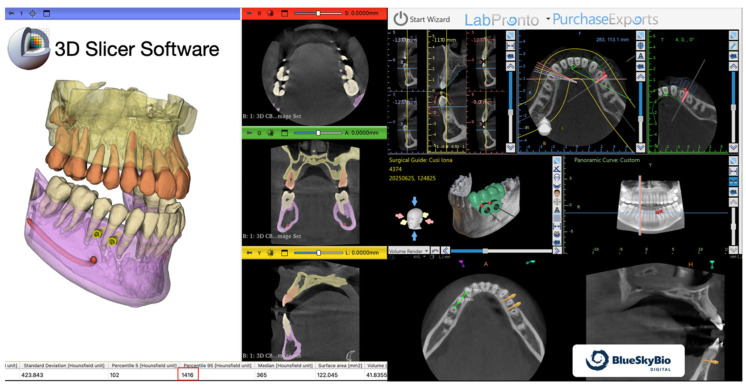
Digital planning software used to assess bone density, evaluate anatomical structures, and assist in miniscrew placement planning.

This digital workflow enabled assessment of bone quality, available bone volume, and interradicular anatomy at the planned insertion sites using 3D Slicer (version 5.2.x; Brigham and Women’s Hospital, Boston, MA, USA; available at https://www.slicer.org; accessed on 1 March 2026) and Blue Sky Plan software (version 4.13.x; Blue Sky Bio, Libertyville, IL, USA; available at https://www.blueskybio.com; accessed on 1 March 2026) (Figure 1), as previously described for guided orthodontic miniscrew placement [15,16].

### 2.3. Surgical Guides and Miniscrew Placement

**Figure 2:**
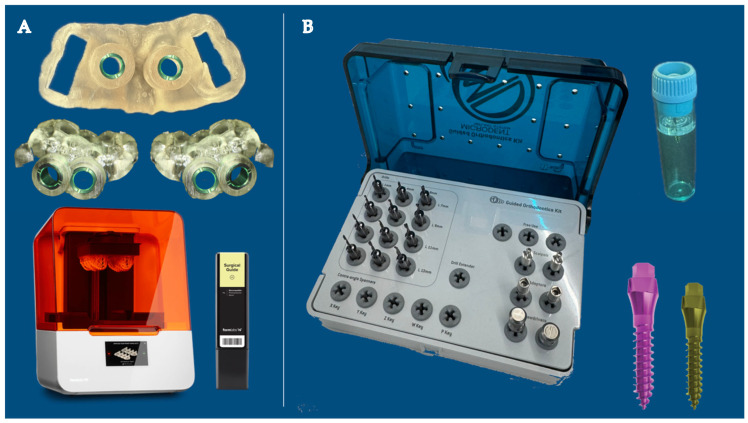
(**A**) Three-dimensional printed surgical guides were designed for each patient—one for the maxilla and two for the mandible—and fabricated using a biocompatible surgical guide resin (Surgical Guide Resin, Formlabs Inc., Somerville, MA, USA). The guides were manufactured using a Formlabs Form 3B 3D printer (Formlabs Inc., Somerville, MA, USA) based on stereolithography (SLA) technology, following the manufacturer’s recommended printing and post-curing protocol. (**B**) Microdent Swift miniscrew and Microdent Orthodontic Guided Surgical Kit were used.

Three-dimensional printed surgical guides were designed for each patient—one for the maxilla and two for the mandible—and fabricated using a biocompatible surgical guide resin (Surgical Guide Resin, Formlabs Inc., Somerville, MA, USA) (Figure 2A). The use of guided surgery aimed to improve placement accuracy and reduce operator-dependent variability, as previously reported in the literature [15,16].

Two palatal miniscrews (diameter × length: 2.4 × 13 mm) were inserted in the paramedian palatal region to provide anchorage for the maxillary appliance, following anatomical recommendations described by Winsauer et al. [16].

Four mandibular miniscrews (diameter × length: 2.0 × 9 mm; two per side) were placed interradicularly between teeth 33–34, 34–35, 43–44, and 44–45, at least 1 mm apical to the mucogingival junction, with an insertion angulation of approximately 20°, to achieve bicortical anchorage and reduce the risk of root contact [17].

Miniscrew insertion was performed under local anesthesia using a guided protocol. In all cases, predrilling was performed according to bone quality, and insertion torque was maintained within the recommended range of 25–35 Ncm for D1–D2 bone, in accordance with previously published biomechanical guidelines [13,14,18]. A guided surgical kit compatible with the guide sleeve and the Swift miniscrew system (Microdent System, Barcelona, Spain) was used (Figure 2B).

### 2.4. Appliance Design and Activation Protocol

Following miniscrew placement, both arches were rescanned with the miniscrews in situ, and a construction bite was recorded to position the mandible in a Class I sagittal relationship. Based on these records, a bone-anchored maxillary appliance (with or without transverse expansion, depending on individual requirements) and two custom-fabricated supragingival stainless-steel mandibular miniplates were manufactured.

Each mandibular miniplate was fixed supragingivally onto the corresponding miniscrews and incorporated a coupling element compatible with a fixed Herbst-type advancement mechanism, similar in principle to previously described bone-anchored functional protocols [7,8,19].

**Figure 3:**
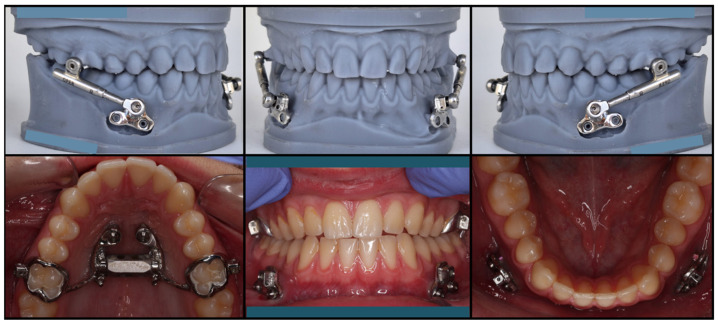
Bone-supported Herbst appliance components. Upper appliance (MAPE-miniscrew-assisted palatal expansion, when expansion was indicated, or bone-supported transpalatal bar) and mandibular supragingival miniplates coupled to the telescopic advancement system (PowerScope 2; American Orthodontics, Sheboygan, WI, USA).

The maxillary and mandibular components were connected using a telescopic Class II advancement device (PowerScope 2; American Orthodontics, Sheboygan, WI, USA). Initial activation was performed at appliance delivery to achieve the planned mandibular advancement corresponding to a Class I sagittal relationship (Figure 3).

**Figure 4:**
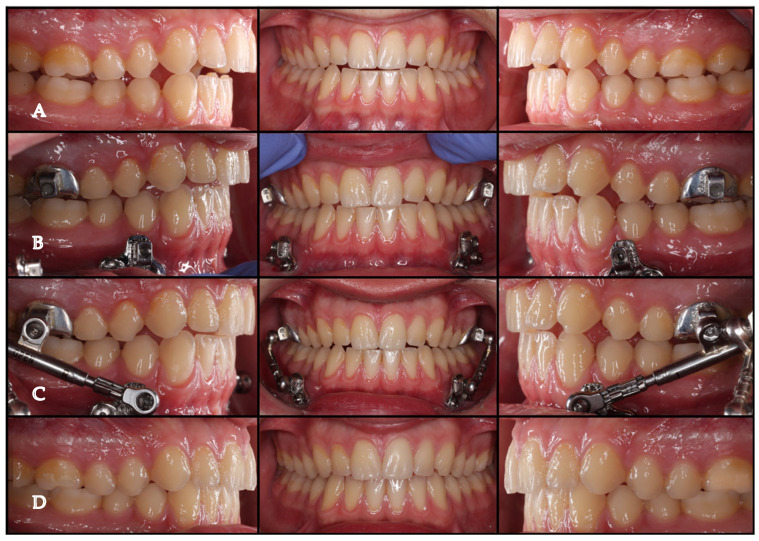
Clinical follow-up of the supragingival bone-supported Herbst protocol: (**A**) Initial intraoral situation and occlusion. (**B**) Intraoral view after appliance placement showing the supragingival design. (**C**) Follow-up during treatment progression. (**D**) Final intraoral situation after completion of the Herbst phase, with correction to a Class I sagittal relationship.

Patients were reviewed at monthly intervals over a 10-month treatment period. Additional activation was performed when necessary to maintain continuous mandibular advancement and compensate for functional adaptation or any residual sagittal discrepancy until a stable Class I relationship was achieved (Figure 4).

The duration of the Herbst phase (10 months) was determined based on clinical criteria, specifically the time required to achieve a stable Class I sagittal relationship under continuous mandibular advancement. This duration is consistent with previously reported protocols in late-adolescent patients.

The Herbst appliance was used as the first phase of a comprehensive orthodontic treatment, which was subsequently followed by fixed appliance therapy to finalize occlusion.

No additional functional appliances were used during the Herbst phase. Maxillary expansion (MAPE) was incorporated only in cases where transverse deficiency was clinically diagnosed.

### 2.5. Cephalometric Analysis

Standardized lateral cephalograms were obtained before treatment (T0) and immediately after completion of the Herbst phase (T1). Cephalometric analysis included conventional sagittal and vertical parameters: SNA, SNB, ANB, Wits appraisal, mandibular plane angle (SN–GoGn), and maxillary and mandibular incisor inclinations. The mandibular plane angle (SN–GoGn) was used to assess vertical skeletal changes and potential mandibular rotation, according to established cephalometric analyses [20,21].

**Figure 5:**
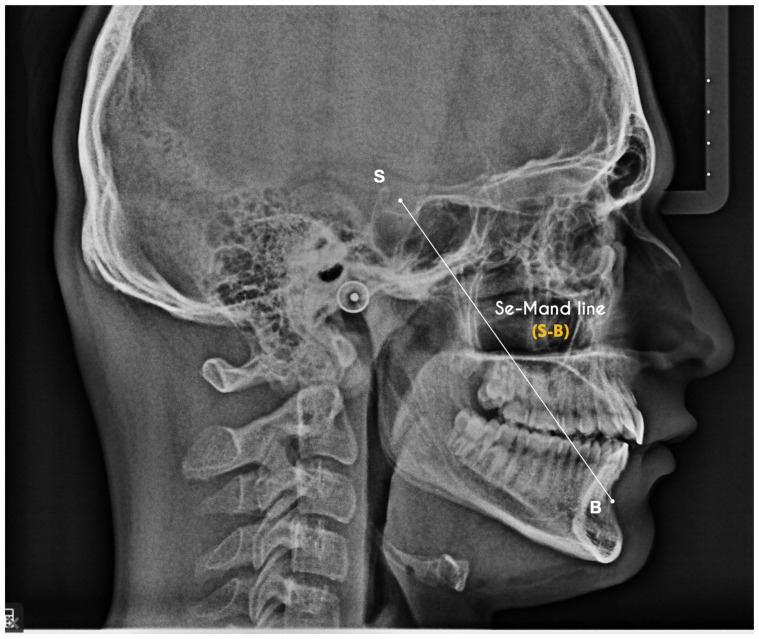
Mandibular effective advancement line (SeMndb-Line). New line of measurement in millimeters to quantify mandibular displacement.

Mandibular effective advancement was additionally assessed using a complementary linear measurement, the SeMndb-line, defined as the linear distance between Sella (S) and point B (Figure 5). This parameter was used to quantify anteroposterior mandibular displacement in millimeters, minimizing the influence of cranial base rotation and vertical skeletal divergence [22,23]. The SeMndb-line was introduced as a complementary linear parameter to quantify effective mandibular advancement in millimeters. This measurement was not intended to replace established angular or appraisal-based cephalometric analyses but to provide an adjunctive linear assessment of sagittal mandibular displacement.

All measurements were performed by a calibrated examiner. To assess measurement reliability, 20% of the radiographs were randomly selected and remeasured after a two-week interval by the same examiner and a second independent examiner. Intra- and interobserver reliability were evaluated using intraclass correlation coefficients (ICC) and Dahlberg’s error.

### 2.6. Statistical Analysis

No a priori sample size calculation was performed because this investigation was designed as a pilot study aimed at estimating effect sizes and feasibility.

Descriptive statistics were calculated for all variables and expressed as mean ± standard deviation. Normality of data distribution was assessed using the Shapiro–Wilk test. For normally distributed variables, paired Student’s *t*-tests were used to compare pre- and post-treatment values; otherwise, the Wilcoxon signed-rank test was applied. Statistical significance was set at *p* < 0.05.

Pearson correlation analysis was performed to explore associations between the SeMndb-line and conventional sagittal indicators (ANB and Wits appraisal). All statistical analyses were conducted using SPSS software (version 26.0; IBM Corp., Armonk, NY, USA).

## 3. Results

All eleven patients completed the 10-month treatment protocol without appliance failure, miniscrew loosening, or loss of skeletal anchorage. The demographic and clinical characteristics of the study sample are summarized in Table 1. No surgical or soft-tissue complications related to miniscrew insertion or supragingival miniplates were observed. Postoperative discomfort was mild and transient in all cases, and no interruptions of treatment were required. These findings confirm the clinical feasibility and short-term stability of the supragingival bone-supported Herbst protocol.

### 3.1. Skeletal Changes

Cephalometric analysis demonstrated a significant improvement in sagittal skeletal relationships following treatment. The SNB angle increased from 75.73° ± 2.72 at baseline to 79.09° ± 1.58 after treatment, corresponding to a mean increase of +3.36° (*p* < 0.001), indicating effective mandibular advancement. In contrast, the SNA angle showed a small, non-significant increase (+1.13°, *p* = 0.099), suggesting minimal maxillary displacement. Detailed pre- and post-treatment cephalometric values and statistical comparisons are reported in Table 2.

As a consequence of these changes, sagittal discrepancy improved substantially. The Wits appraisal decreased from +3.00 ± 1.34 mm to +0.35 ± 0.85 mm, with a mean reduction of −2.65 mm (*p* < 0.001), reflecting correction toward a near-Class I skeletal relationship. No clinically relevant changes were observed in the mandibular plane angle (SN–GoGn) (Table 2), indicating that no clinically relevant vertical mandibular rotation occurred.

**Figure 6:**
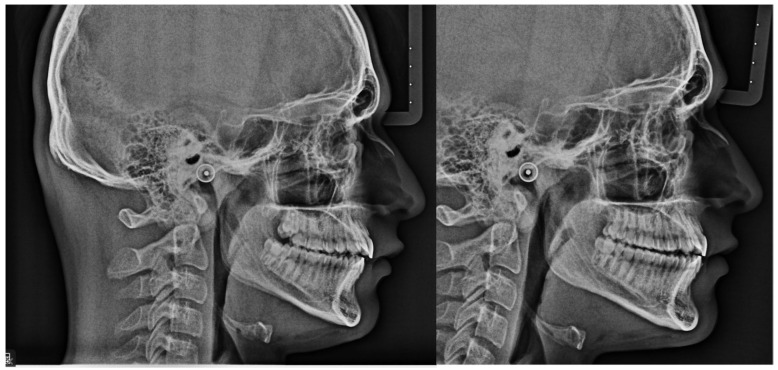
Lateral radiographs before and after treatment with the supragingival miniplate (SGMP)-supported Herbst device.

**Figure 7:**
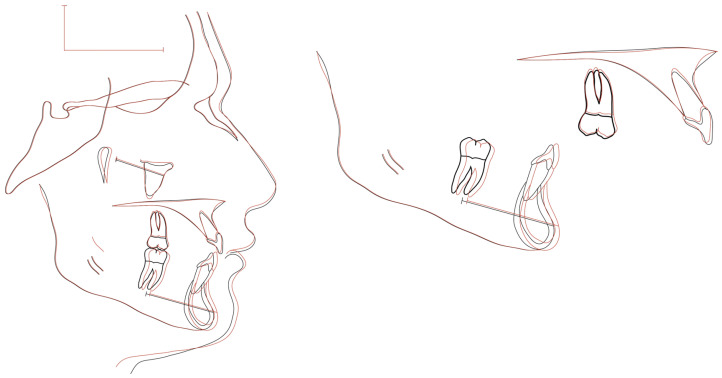
The 2D superimpositions where skeletal changes are observed.

Mandibular effective advancement, assessed using the complementary linear SeMndb-line (Sella–B), increased significantly from 118.97 ± 1.97 mm to 122.46 ± 1.76 mm, corresponding to a mean advancement of +3.49 mm (*p* < 0.001). This linear measurement confirmed the magnitude of sagittal mandibular displacement observed with angular parameters (Figure 6 and Figure 7).

These findings suggest that the observed skeletal changes are not only statistically significant but also clinically relevant in the context of Class II correction in late adolescents.

### 3.2. Dentoalveolar Changes

**Figure 8:**
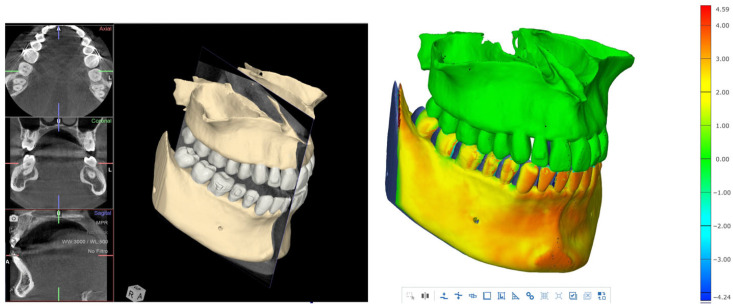
The 3D superimpositions where skeletal changes are observed.

Dentoalveolar effects were minimal. Lower incisor inclination remained essentially unchanged throughout treatment, with a mean variation of −0.18° (*p* = 0.909), indicating excellent control of mandibular incisor position and absence of systematic proclination. Upper incisor inclination showed only minor changes, with no clinically relevant dental compensation. These findings suggest that the applied forces were predominantly absorbed by the skeletal anchorage rather than transmitted to the dentition (Figure 6, Figure 7 and Figure 8).

### 3.3. Correlation Analysis

Pearson correlation analysis revealed strong and statistically significant negative associations between mandibular effective advancement measured by the SeMndb-line and conventional sagittal indicators. Higher SeMndb values correlated with lower ANB angles (r = −0.78, *p* = 0.004) and lower Wits appraisal values (r = −0.81, *p* = 0.002), confirming the consistency between the linear and angular assessments of skeletal Class II correction. Pearson correlation coefficients between SeMndb-line and sagittal indicators are shown in Table 3.

### 3.4. Reliability Analysis

Measurement reliability was high. Intra- and interobserver intraclass correlation coefficients for all cephalometric variables exceeded 0.90, indicating excellent reproducibility. Dahlberg error values were low and within clinically acceptable limits, confirming the robustness of the measurement protocol.

## 4. Discussion

The present pilot clinical study evaluated a minimally invasive supragingival bone-supported Herbst protocol combined with a fully digital, guided workflow in late-adolescent patients with skeletal Class II malocclusion. The main finding was that this approach produced clinically meaningful mandibular advancement with minimal dentoalveolar side effects, particularly effective control of lower incisor inclination, while demonstrating high short-term stability and clinical feasibility.

### 4.1. Skeletal Effects and Biomechanical Considerations

The significant increase in SNB and the reduction in Wits appraisal indicate a substantial improvement in sagittal skeletal relationships. The magnitude of mandibular advancement observed in this study is consistent with previous reports on conventional Herbst therapy [3,6,24], although traditional protocols typically show a higher proportion of dentoalveolar compensation, particularly in late adolescents [3]. These findings support the concept that skeletal anchorage allows functional forces to be transmitted more directly to the basal bone, thereby enhancing the orthopedic component of correction [5,6].

Unlike dentally anchored Herbst mechanics, which are frequently associated with combined skeletal changes, vertical mandibular rotation, and dentoalveolar compensation [25], the present protocol resulted predominantly in horizontal mandibular advancement without clinically relevant vertical changes. This pattern is consistent with previous reports indicating that skeletal anchorage-assisted functional appliances improve force transmission to the basal bone and reduce unwanted dental effects [5,6,26].

### 4.2. Dentoalveolar Control and Comparison with Previous Protocols

One of the most clinically relevant findings was the stability of lower incisor inclination. Lower incisor proclination is a well-documented and often unavoidable side effect of conventional Herbst treatment, particularly in late adolescents [3]. The negligible changes observed in the present sample confirm that supragingival bone-supported anchorage effectively controlled this unwanted effect.

Previous studies combining Herbst appliances with skeletal anchorage have reported improved dental control [5,6]; however, most protocols rely on submucosal miniplates requiring flap elevation and fixation beneath the soft tissues [9,10,11,19]. Although these approaches have demonstrated favorable orthopedic outcomes, they are associated with increased surgical morbidity, postoperative discomfort, and a higher risk of soft-tissue complications [9,11]. In contrast, the supragingival configuration used in this study avoided submucosal plate placement while maintaining anchorage stability under continuous functional loading. The absence of miniscrew loosening or plate-related complications further supports the mechanical reliability of this less invasive design.

### 4.3. Role of Digital Planning and Guided Insertion

The favorable clinical outcomes observed may also be attributed to the use of a fully digital planning and guided insertion workflow. Three-dimensional assessment of bone quality, interradicular space, and anatomical constraints using CBCT and intraoral scanning allows accurate selection of insertion sites and angulation [10,15,16]. Previous studies have demonstrated that guided miniscrew placement improves accuracy and reduces operator-dependent variability, particularly in anatomically demanding regions [10,15,16]. These advantages are especially relevant in complex anchorage systems involving multiple miniscrews, as used in the present protocol.

### 4.4. Complementary Assessment of Mandibular Advancement

Mandibular advancement was additionally explored using the SeMndb-line, a complementary linear measurement between Sella and point B. This parameter was introduced as an adjunct to conventional sagittal indicators, such as ANB angle and Wits appraisal, and was not intended to replace established cephalometric analyses. The strong correlations observed between SeMndb-line and both ANB and Wits appraisal support its internal consistency and suggest its potential usefulness as an adjunctive descriptor of sagittal skeletal changes [22,23].

Nevertheless, this parameter should be interpreted as exploratory and hypothesis-generating. Further validation in larger controlled samples is required before it can be recommended for routine clinical or research use.

### 4.5. Clinical Implications

From a clinical perspective, the supragingival bone-supported Herbst protocol demonstrated high feasibility and favorable patient tolerance. No anchorage failures or appliance-related complications were observed, and postoperative discomfort was mild and transient, consistent with previous reports on minimally invasive skeletal anchorage systems [20,23]. The supragingival design facilitates oral hygiene and simplifies appliance management, which may further enhance patient acceptance and compliance.

This approach may therefore represent a clinically relevant alternative within the spectrum of Class II treatment modalities, particularly in late-adolescent patients, where conventional dentally anchored approaches are often limited and more invasive surgical options may be considered.

To contextualize these findings, a comparative overview of conventional and bone-anchored Herbst protocols is presented in Table 4.

From a practical perspective, patient adaptation to the appliance was rapid, with only mild and transient discomfort reported during the initial days following placement. The supragingival design facilitated oral hygiene and reduced soft-tissue irritation compared with submucosal systems.

Clinically, precise positioning of the miniplates and accurate fit of the coupling elements were essential to ensure stability and avoid mechanical complications. No difficulties were encountered during placement when following the digitally guided protocol, which may reduce operator dependency and improve reproducibility.

These observations may be of particular relevance for clinicians considering the implementation of minimally invasive skeletal anchorage systems in routine practice.

## 5. Limitations and Future Directions

Several limitations must be acknowledged. The sample size was limited, and no control group was included, restricting the generalizability of the findings and preventing direct comparison with conventional or alternative treatment modalities. In addition, the observation period was limited to the active treatment phase, and long-term stability could not be evaluated.

Furthermore, SeMndb-line represents a newly proposed linear descriptor of mandibular advancement, and its clinical applicability and reproducibility should be further investigated in larger samples and comparative study designs.

Future prospective controlled studies with larger samples, extended follow-up, and three-dimensional assessment of mandibular and condylar remodeling using cone-beam computed tomography or other imaging modalities are required to further validate these findings [27,28].

Therefore, the results should be interpreted with caution, as the limited sample size may reduce the statistical power and generalizability of the findings.

## 6. Conclusions

Within the limitations of this pilot clinical study, the supragingival bone-supported Herbst protocol demonstrated high clinical feasibility and produced clinically meaningful sagittal skeletal correction in late-adolescent patients with Class II malocclusion due to mandibular retrusion.

The protocol achieved effective mandibular advancement while maintaining excellent control of dentoalveolar side effects, particularly lower incisor inclination, supporting the role of skeletal anchorage in enhancing the orthopedic component of treatment.

The combination of supragingival miniplates with a fully digital, guided insertion workflow allowed for accurate miniscrew placement, stable anchorage under functional loading, and reduced surgical invasiveness compared with conventional submucosal miniplate systems.

These findings suggest that this approach may represent a minimally invasive and clinically relevant alternative for the treatment of skeletal Class II malocclusion in late adolescents.

However, given the limited sample size, the absence of a control group, and the short-term observation period, further controlled studies with larger samples and long-term follow-up are required to confirm these results.

## Figures and Tables

**Table 1:** Demographic and clinical characteristics of the study sample. The study sample included 11 late-adolescent patients with skeletal Class II malocclusion due to mandibular retrusion. Values are reported as mean ± standard deviation or number and percentage, as appropriate. All patients presented skeletal Class II malocclusion due to mandibular retrusion and fulfilled the inclusion criteria regarding skeletal maturation stage, bone quality, and interradicular space for miniscrew placement.

Variable	n (%) or Mean ± SD	Range
**Age (years)**	15.5 ± 1.2	14–17
**Sex**		
*Male*	5 (45.45%)	—
*Female*	6 (54.55.%)	—
**Skeletal pattern**		
*Mesocephalic (MESO)*	6 (54.5%)	—
*Brachycephalic (BRAQUI)*	3 (27.3%)	—
*Dolichocephalic (DOLICO)*	2 (18.2%)	—

**Table 2:** Descriptive statistics of cephalometric variables before (T0) and after (T1) treatment with the supragingival bone-supported Herbst appliance. Mean values, standard deviations, mean differences (Δ), and results of paired statistical comparisons are reported.

Variable	Mean Pre ± SD	Mean Post ± SD	Mean Difference (Δ)	t	*p*-Value	Significance
**SNA (°)**	80.36 ± 2.58	81.50 ± 1.36	+1.13	1.82	0.099	n.s.
**SNB (°)**	75.73 ± 2.72	79.09 ± 1.58	+3.36	6.00	0.0001	Significant
**Lower incisor inclination (°)**	23.18 ± 6.15	23.00 ± 1.84	−0.18	−0.12	0.909	n.s.
**SeMndb-line (mm)**	118.97 ± 1.97	122.46 ± 1.76	+3.49	7.04	0.00004	Significant
**Wits (mm)**	3.00 ± 1.34	0.35 ± 0.85	−2.65	−5.23	0.0004	Significant

Note: Paired Student’s *t*-test; statistical significance set at *p* < 0.05. Abbreviations: n.s., not significant. To assess measurement reliability, two independent observers retraced 20% of the radiographs after two weeks. The Dahlberg error and intra-/interobserver ICCs were calculated for all cephalometric variables (ICC > 0.90), confirming excellent reproducibility. Additional statistical analysis confirmed normal data distribution (Shapiro–Wilk test, *p* > 0.05); therefore, paired *t*-tests were applied. Significant skeletal improvements were found in SNB (Δ = +3.36° ± 0.6°, *p* < 0.001) and SeMndb-line (Δ = +3.49 ± 0.9 mm, *p* < 0.001), indicating effective mandibular advancement. The SNA angle demonstrated a non-significant increase (+1.13° ± 1.2°, *p* = 0.099). Wits appraisal improved significantly (Δ = −2.65 ± 0.7 mm, *p* < 0.001). Lower incisor inclination remained stable, with a mean variation of −0.18° (*p* = 0.909), indicating the absence of consistent proclination.

**Table 3:** Pearson correlation coefficients between mandibular effective advancement measured by the SeMndb-line and conventional sagittal skeletal indicators (ANB angle and Wits appraisal).

Variable Pair	r	*p*-Value
SeMndb vs. ANB	−0.78	0.004
SeMndb vs. Wits	−0.81	0.002

Note: Negative correlation coefficients indicate greater mandibular advancement associated with improved sagittal skeletal relationships.

**Table 4:** Comparative overview of anchorage strategies in Herbst therapy.

Aspect	Conventional Dentally Anchored Herbst	Submucosal Bone-Anchored Herbst (Miniplates)	Supragingival Bone-Supported Herbst (Present Protocol)
**Anchorage type**	Dental anchorage (bands, crowns)	Skeletal anchorage via submucosal miniplates	Skeletal anchorage via supragingival stainless-steel miniplates fixed to miniscrews
**Invasiveness**	Non-surgical	Surgical flap elevation required	Minimally invasive, no flap elevation
**Placement technique**	Conventional laboratory and chairside procedures	Surgical placement under mucosal flaps	Digitally planned CBCT + STL workflow with 3D-printed surgical guides
**Skeletal effect**	Moderate; partially masked by dentoalveolar compensation	High; effective force transfer to basal bone	High; clinically meaningful mandibular advancement observed
**Lower incisor proclination**	Frequent and clinically relevant	Minimal	Negligible (Δ = −0.18°, *p* = 0.909)
**Anchorage loss**	Common in late adolescents	Rare	None observed in this pilot study
**Patient morbidity**	Low	Moderate (postoperative discomfort, soft-tissue risk)	Low; mild and transient discomfort only
**Soft-tissue complications**	Appliance-related irritation possible	Higher risk (inflammation, plate exposure)	None observed
**Appliance management**	Relatively simple	More complex due to surgical component	Simplified access and hygiene due to supragingival design
**Suitability for late adolescents**	Limited by anchorage loss	Effective but more invasive	Well suited; balances skeletal efficacy and reduced morbidity
**Evidence level**	Extensive literature	Growing but limited	Pilot clinical evidence (present study)

## Data Availability

The data presented in this study are available upon reasonable request from the corresponding author.
